# Perspective: Use of protein S100B as a quality assurance marker for endovascular therapy in acute ischemic stroke

**DOI:** 10.3389/fneur.2025.1488018

**Published:** 2025-02-04

**Authors:** Franziska Lieschke, Christian Foerch

**Affiliations:** ^1^Department of Neurology, University Hospital, Goethe University Frankfurt, Frankfurt/Main, Germany; ^2^Department of Neurology, RKH klinikum Ludwigsburg, Ludwigsburg, Germany

**Keywords:** quality assurance, biomarker, S100B, acute ischemic stroke, mechanical thrombectomy

## Abstract

Mechanical thrombectomy (MT) is a highly effective treatment for ischemic stroke associated with large vessel occlusion. Given its complexity, this procedure is widely used throughout the world in hospitals with different levels of experience. Therefore, practical quality assurance is advised to ensure a high standard of care across the board. In this perspective article, we propose the implementation of measuring serum S100B after MT as a surrogate outcome parameter for the extent of tissue damage as an additional quality indicator for internal and external benchmarking in endovascular therapy. We focus on the analysis of patients, in whom there is a discrepancy between the expected (e.g., based on favorable preconditions) and the actual biomarker outcome. We aim to illustrate the advantages and drawbacks of measuring S100B after MT, reliably depicting the procedure’s quality and its use for comparison and identification of “outlier” patients in MT patient cohorts for further process and single-case analysis.

## Introduction

Mechanical thrombectomy (MT) is a highly effective treatment for ischemic stroke associated with large vessel occlusions (LVOs) (1–4). However, it is a resource-intensive procedure from a logistical and technical point of view, as it frequently requires careful patient selection with advanced imaging techniques, inter-hospital transfers, and post-procedural care in the neurological intensive care unit. Furthermore, trained interventionalists within a multidisciplinary team and appropriate devices are needed. Simple, practicable quality assurance is important to maintain high standards within and across different centers. In particular, differentiation between directly associated complications (i.e., unsuccessful MT or reperfusion injury) and indirectly associated complications (i.e., pneumonia after endotracheal intubation, status epilepticus, and cardiac complications) is warranted. At present, the “modified thrombolysis in cerebral infarction” (mTICI) score as a measure for recanalization success during angiography and the short-term case fatality rate are often used as benchmarks (5, 6), but these have their limitations, particularly with regard to differentiating the above-mentioned causes of poor outcomes.

## Serum protein S100B levels in acute ischemic stroke

We recently demonstrated that serum levels of the astroglial protein S100B reliably indicate the extent of ischemic tissue damage after MT and suggested its use as a surrogate outcome parameter, providing added value in clinical routine and interventional trials (7). S100B concentrations can be rapidly assessed using electrochemiluminescence immunoassay techniques (8). Elevated S100B serum concentrations measured 48–72 h after symptom onset are highly correlated with final infarct volume (the infarct core) and functional outcome (9–11). In contrast, large perfusion deficits that do not transition into a demarcated infarction (e.g., due to successful MT) do not cause the release of S100B into the serum (12). Any complication resulting in additional brain tissue damage likely causes S100B release (e.g., intracerebral or subarachnoid hemorrhage, arterial re-occlusion, and hypoxia due to extracerebral complications) (10, 13–16).

## Proposed use of serum S100B as a quality assurance indicator

We previously observed that especially “successful” recanalization (mTICI >2) resulted in both low and high S100B levels. There are various possible explanations for this, which is why the implementation of S100B in a quality monitoring system is of particular interest.

**Figure 1:**
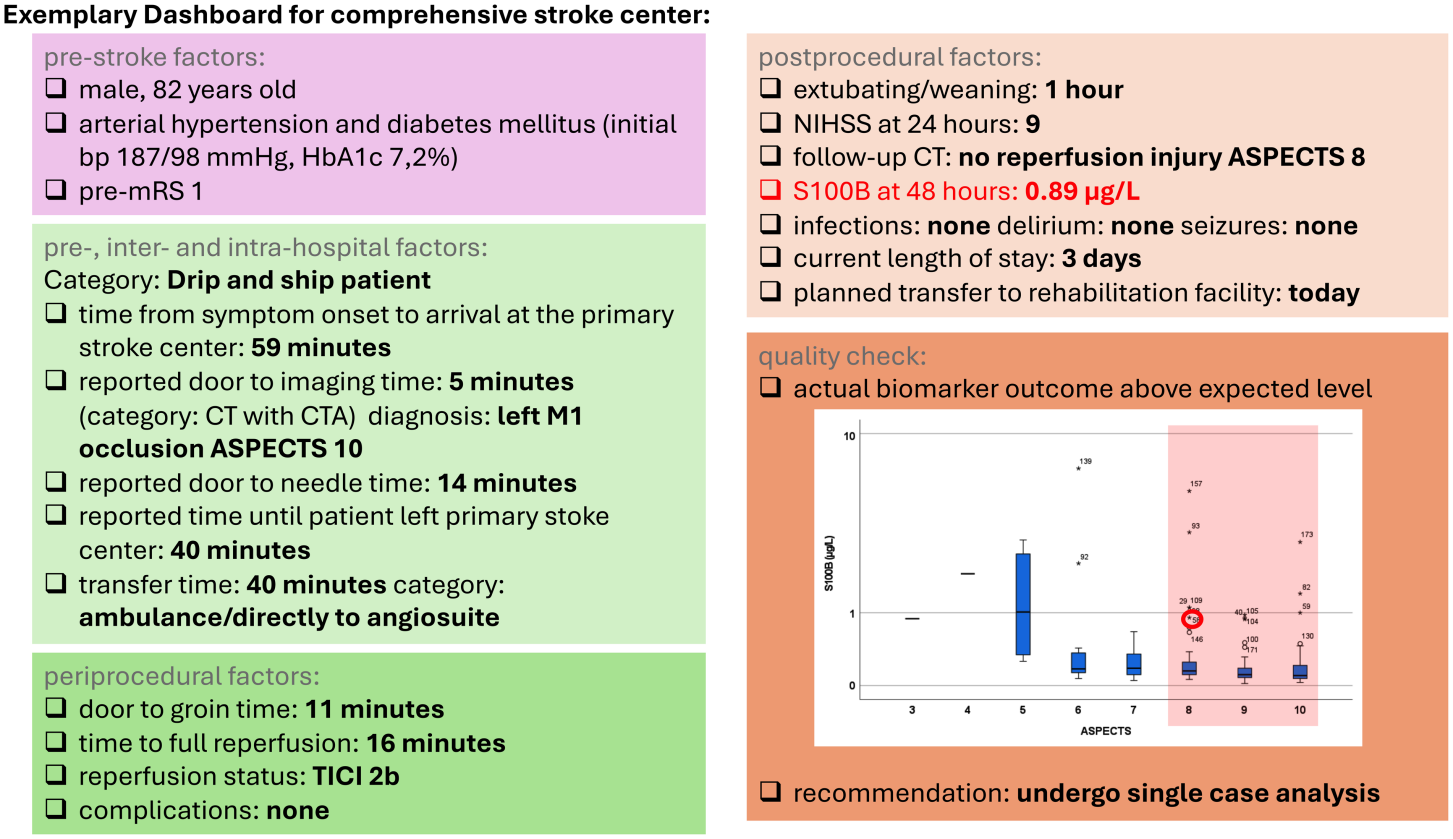
Exemplary dashboard for a comprehensive stroke center. Boxplots depict the distribution of the S100B values stratified according to the Alberta Stroke Program Early CT Score (ASPECTS). Highlighted S100B outliers and extreme values were used for the following analysis. mRS, modified Rankin Scale; CT, computed tomography; CTA, computed tomography angiography; ASPECTS, Alberta Stroke Program Early CT Score; TICI, thrombolysis in cerebral infarction; NIHSS, National Institutes of Health Stroke Scale.

The determination of S100B after MT can be used for performance feedback to healthcare professionals, in addition to data obtained from regular quality registries. As such, it can be easily integrated as an outcome indicator into dashboards [as currently being investigated in the PERFEQTOS performance feedback trial (17)]. This approach can be incorporated into existing treatment concepts without requiring additional resources (Figure 1).

To assess the utility of S100B as a quality indicator for endovascular therapy, we measured S100B levels in patients’ serum on the second day after MT for over a year and analyzed the values together with our existing data (7). Detailed information on the methods can be found in the Supplementary material.

We examined 183 patients with acute middle cerebral artery infarction associated with LVO, of whom 44% were female. The mean age of the patients was 69.2 ± 13.6 years. The median Alberta Stroke Program Early CT Score (ASPECTS) on the first CT scan was 8 (IQR 7–10). The median S100B level in the entire cohort was 0.15 μg/L (IQR 0.09–0.32 μg/L), the S100 B values in the first tertile were below 0.103 μg/L, the values in the second tertile ranged between 0.103 and 0.229 μg/L, and the values in the third tertile were above 0.229 μg/L. S100B levels showed a high correlation with infarct size (Spearman *r* = 0.74, *p* < 0.001). Successful recanalization (mTICI ≥2) was achieved in 95% of cases in the first S100B tertile, 92% in the second S100B tertile, and 73% in the third S100B tertile. A good functional outcome at discharge (mRS ≤ 2) was achieved in 72% of patients in the first tertile, 49% in the second tertile, and 18% in the third tertile. Patients with successful recanalization (mTICI ≥2b, *n* = 159) showed on average significantly smaller infarcts (37.9 mL ± 65.4 mL vs. 137.2 mL ±132.4 mL *p* < 0.001) and lower S100B concentrations (median 0.13 μg/L vs. 0.44 μg/L *p* < 0.001) than patients in whom recanalization was unsuccessful (mTICI<2b, *n* = 19).

### Example 1: high S100B levels after MT despite high ASPECTS

We further analyzed patients who showed a discrepancy between the expected (based on given conditions) and the actual biomarker outcome. All patients with S100B upper outliers (1.5–3x the IQR) and upper extreme values (>3x the IQR) indicating significant brain tissue destruction despite an ASPECTS of 8–10 at admission were analyzed for underlying factors (e.g., futile recanalization and complicative intracranial hemorrhage). We identified 129 patients with this favorable precondition, and the median S100B value in this subgroup was 0.12 μg/L (0.08–0.23 μg/L). For this group, an acceptable (‘expected’) biomarker outcome would range from the lower level of detection up to a maximum of 0.345 μg/L. We further selected 16 patients with S100B values above this limit to undergo single-case analysis. Futile recanalization was found in three patients with mTICI 0 and two patients with mTICI 2a (due to pre-existing stenosis of the middle cerebral artery (MCA) and calcified emboli, respectively). One patient hereof developed a malignant infarction. Eight patients had partially successful recanalization (mTICI 2b) with peripheral artery branch occlusions described in three patients, hemorrhagic transformation in four patients (including three patients with parenchymal hemorrhage), and development of a malignant infarction in one patient. Two patients were treated beyond a 6-h time window. Three patients had complete recanalization (mTICI 3) but developed significant infarcts and one with HT. When comparing the 16 “outlier” patients to all other patients with an initial ASPECTS of 8–10, recanalization rates differed significantly toward a higher percentage of futile MT (mTICI 0 in 19% vs. 4%) and lower rates of successful recanalization (mTICI 3 or 2b in 19% vs. 38, 50% vs. 56% *p* = 0.007). Accordingly, “outlier” patients yielded significantly increased National Institutes of Health Stroke Scale (NIHSS) scores at 24 h (median of 24 vs. 6 points, *p* < 0.001) and infarct volumes (mean 146.2 ± 105.2 mL vs. 14.5 ± 22.6 mL, *p* < 0.001). The hemorrhagic transformation occurred in 38% of the outliers, whereas only 24% of all other patients with an initial ASPECTS of 8–10 developed HT.

### Example 2: unfavorable outcomes despite a low S100B

In the second example, we looked at patients having an unfavorable clinical outcome (modified Rankin Scale “mRS” 4–6) at discharge despite very low S100B levels (i.e., little tissue damage). We identified 60 patients with serum S100B values below the upper normal range of 0.105 μg/L, of whom 11 still had an unfavorable clinical outcome. In addition, we aimed to characterize the responsible factors (e.g., extracerebral complications after successful MT). In all of these patients, recanalization was successful (mTICI 2b, 2c, 3), and only small infarct volumes were measured (mean 5.7 ± 7.8 mL vs. 100.3 ± 111.1 mL in all other patients with mRS 4–6). In three patients, CT hyperdensities were described; however, differentiation of iodine contrast staining vs. hemorrhagic transformation was not possible. Ten of eleven patients developed pneumonia, five of whom were refractory to treatment after failing multiple antibiotic therapy regimes. The infection resulted in death in three of these patients. Furthermore, we found frequent constellations of high age and (pre-) existing comorbidities, which were acutely exacerbated. As such, four patients developed acute cardiac complications based on previous chronic heart disease. Altogether, we identified respiratory and weaning failures as one of the main reasons for unfavorable outcomes despite successful MT (*n* = 6). Two patients suffered early recurrent strokes, which occurred after day 2 (and therewith after the first follow-up imaging used for evaluation of the infarct volume and the S100B collection on day 2), of whom one patient suffered additional epileptic seizures. Three patients failed to achieve a symptomatic recovery in the absence of relevant tissue damage.

## Discussion

To identify emerging shortcomings in the quality of care early ahead, the practice of quality assurance, especially in hospitals with limited experience, is of utmost relevance. In our opinion, protein S100B as a measure of ischemic brain tissue damage (i.e., infarct volume) is a feasible quality assurance marker. By introducing S100B measurements into our quality monitoring system, we identified futile recanalization and hemorrhagic complications as the main reasons for noticeably increased S100B levels despite successful recanalization in our MT cohort. A frequent reason for poor functional outcome at discharge despite low S100B values was post-stroke complications such as infections, especially aspiration pneumonia after endotracheal intubation for MT, or exacerbation of pre-existing disorders.

Determination of S100B is objective and readily available, thereby reliably indicating brain tissue outcomes after MT. In contrast to the mTICI score measuring the recanalization success during angiography, no further special expertise is necessary for its application. Moreover, mTICI is determined during angiography, and re-occlusions that occur after the completion of the intervention—contributing to infarct development despite initially restored flow—cannot be captured by this measure. Using the short-term case fatality rate for monitoring and benchmarking the quality of MT can be misleading, as this metric does not account for lethal complications that may occur despite a successful MT procedure. In this study, the distinction of whether these complications are related to the intervention can be easily made by determining S100B, which highlights its particular importance for quality assessment. Establishing the monitoring of MT by S100B at a center does not require any special resources other than those available at an average hospital of standard care. The determination of S100B can be easily integrated into the routine care of a stroke unit, with a single blood draw 48–72 h after MT (10) in all MT patients. By looking at the individual S100B value distribution during 1 year and single-case analysis of outliers as well as comparison of the distribution by benchmark with other hospitals and previous years, changes in complication rates, unusually high mortality rates (not directly associated with the stroke or MT), and trends toward improvement and deterioration of procedure quality can be detected early. We confirmed that S100B levels in our cohort correlated highly significantly with infarct demarcation on follow-up imaging. The non-invasive determination by blood test, due to the lack of radiation exposure, lower costs, and its point of care (POC) character, may represent a decisive advantage over follow-up imaging, especially in situations of limited resources.

Particularly after general anesthesia is required for MT, the clinical assessment of the stroke severity may be clouded by concomitant extracerebral diseases and complications. Thus, S100B may offer additional diagnostic value, especially in cases where neuroimaging is not immediately conclusive or in the hyperacute stage before significant radiographic changes are visible. Across multiple studies, adding S100B to clinical and imaging assessments results in improved AUROCs, indicating a moderate but real enhancement in predictive accuracy (14, 18–20). In this regard, the study of Honegger et al. is of particular interest, where using serum S100B added to the pre-defined prediction models led to an increase in the AUC and reclassification indices, showing its value beyond the established risk factors (20).

Other brain-specific biomarkers used following stroke include neuron-specific enolase (NSE) (21), tau (22, 23), neurofilament light chain (NfL) (24, 25), glial fibrillary acidic protein (GFAP) (26–28), and ubiquitin C-terminal hydrolase L1 (UCH-L1) (27, 28). Despite showing great potential in stroke diagnostics and prognosis, they are not yet widely used in routine clinical practice (29). Their benefits are obvious. As such, they can help differentiate between ischemic and hemorrhagic strokes (especially GFAP), provide early detection of neuronal or glial damage, and offer insights into patient prognosis (7, 14, 30, 31). Second, they can be used to select and adapt therapies in terms of personalized medicine, reducing unnecessary, expensive tests and procedures (12). In addition, they can be used to assess the risk of developing certain complications, which can influence the decision to take preventive measures (13, 20). However, challenges remain. A lack of standardized protocols and threshold values limits their broader use. Some lack specificity and can be elevated in other neurological or systemic conditions, leading to potential misinterpretation (32). Alarmins and cell-free DNA (cfDNA) have also emerged as promising candidates. Frequent reviews here raise the question of whether cfDNA is merely a marker of severe brain injury released from damaged cells or an active contributor to brain injury or stroke-associated infection. In this study, the particular source of cfDNA may be important to consider as they may have different functional properties and whether it is released from necrotic lysed cells or actively expelled from cells (33–35).

There are several limitations to our study. First, this study does not capture patients who have experienced time delays when being transferred from primary stroke centers, received repeated imaging on arrival to our center (usually MR-guided, bearing the risk for further time delays), and were excluded from MT based on an extending ischemic demarcation evident on the very same imaging. Second, to ensure the proper identification of explanatory factors and to determine the most likely applicable causalities, prior experience in the evaluation of medical findings must be available. This requires either a certain level of training for the evaluating person from quality assurance or the use of, e.g., computer-assisted analysis, which in turn requires advanced and precise data collection from the clinics. However, this should not add to the already existing, in part extremely extensive, documentation obligations of medical and nursing staff.

We demonstrate the feasibility and limitations of using S100B in the quality analysis after MT. Measuring S100B after MT can reliably depict the procedure’s quality, allowing comparison and identification of “outlier” patients in MT patient cohorts for further process evaluation and single-case analysis.

## Data Availability

The raw data supporting the conclusions of this article will be made available by the authors, without undue reservation.
