# Supplementary material for: Perspective: Use of protein S100B as a quality assurance marker for endovascular therapy in acute ischemic stroke
Source: Front Neurol. 2025 Feb 4;16:1488018. doi: 10.3389/fneur.2025.1488018 (PMC11832400; doi:10.3389/fneur.2025.1488018)
Supplement: Supplementary file 1 [file Data_Sheet_1.PDF]

Methods (original data)

From January to December 2021, consecutive patients admitted to our comprehensive stroke center with an acute middle cerebral artery (MCA) infarction due to large vessel occlusion (LVO) subjected to undergo mechanical thrombectomy (MT) were included. Patients younger than 18 years were excluded, as well as patients with any stroke or transient ischemic attack (TIA) within the last 3 months. Moreover, known malignant melanoma in medical history and traumatic brain injury within the last 3 months (including head concussion at symptom onset) were further exclusion criteria due to known increase of S100B concentration in these conditions[1,2]. The data was pooled with data from our previous study “The Biomarker for Prediction of Outcome After Mechanical Thrombectomy in Acute Ischemic Stroke Study” (BE PROMETHEUS)[3] to permit time trends and comparison over several years. The study was approved by the local ethics committee (No. 242-17). Written informed consent for study participation was obtained from patients or their legal representatives.

Clinical baseline parameters were captured as follows: age, sex, modified Rankin scale (mRS) before admission, information on previous diseases and vascular risk factors, National Institutes of Health Stroke Scale (NIHSS) at admission and at 24 hours, stroke etiology according to Trial of Org 10172 in Acute Stroke Treatment (TOAST) classification, treatment with intravenous thrombolysis, duration between symptom onset, arrival in hospital, and endovascular treatment, Alberta Stroke Program Early CT Score (ASPECTS), mTICI score as well as peri-interventional complications (such as infections, hemorrhagic transformation or cardio-vascular events). Quantitative volumetry was performed on follow-up brain imaging (usually cranial computer tomography scan at day 1 after intervention) with Image J® to determine final infarct volume after thrombectomy. Three months after MT, a

standardized telephone follow-up interview was performed with each patient or their relatives to determine the current mRS.

We collected the venous blood samples on day two after MT, as this time point showed the best correlation with final infarct volume and outcome after MT in previous studies[4–7]. S100B was determined using commonly available ECLIA techniques (Elecsys S100, Roche Diagnostics Deutschland GmbH, Mannheim, Germany)[8]. The technical details were as followed: lower limit of detection 0.005 µg/L, measuring range 0.005 – 39 µg/L, repeatability 0.7-2.1%, reproducibility 1.7-3.1%, 95% percentile of normal controls 0.105 µg/L.

Statistical analyses were performed using IBM® SPSS® Statistics, Version 22 (Statistical Package for the Social Sciences, Armonk, NY, USA). Baseline variables and S100B values were compared between the S100B tertile groups using t test, Mann Whitney U test, Kruskal-Wallis-test or χ^2 test, depending on the level of measurement. A significance level of alpha = 0.05 was chosen for all tests. We analyzed S100B outliers (S100B values of 1.5 - 3x the interquartile range, IQR) and extreme values (>3x the IQR).

1. Goyal A, Failla MD, Niyonkuru C, et al. S100b as a prognostic biomarker in outcome prediction for patients with severe traumatic brain injury. *J Neurotrauma*. 2013;30(11):946-957.
2. Harpio R, Einarsson R. S100 proteins as cancer biomarkers with focus on S100B in malignant melanoma. *Clin Biochem*. 2004;37(7):512-518.
3. Luger S, Koerbel K, Oeckel AM, Schneider H. Role of S100B Serum Concentration as a Surrogate Outcome Parameter After Mechanical Thrombectomy. 2021;0.
4. Büttner T, Weyers S, Postert T, Sprengelmeyer R, Kuhn W. S-100 protein: serum marker of focal brain damage after ischemic territorial MCA infarction. *Stroke*. 1997;28(10):1961-1965.
5. Foerch C, Singer OC, Neumann-Haefelin T, du Mesnil de Rochemont R, Steinmetz H, Sitzer M. Evaluation of serum S100B as a surrogate marker for long-term outcome and infarct volume in acute middle cerebral artery infarction. *Arch Neurol*. 2005;62(7):1130-1134.

6. Missler U, Wiesmann M, Friedrich C, Kaps M. S-100 protein and neuron-specific enolase concentrations in blood as indicators of infarction volume and prognosis in acute ischemic stroke. *Stroke*. 1997;28(10):1956-1960.
7. Herrmann M, Vos P, Wunderlich MT, de Bruijn CH, Lamers KJ. Release of glial tissue-specific proteins after acute stroke: A comparative analysis of serum concentrations of protein S-100B and glial fibrillary acidic protein. *Stroke*. 2000;31(11):2670-2677.
8. Mussack T, Kirchhoff C, Buhmann S, et al. Significance of Elecsys S100 immunoassay for real-time assessment of traumatic brain damage in multiple trauma patients. *Clin Chem Lab Med*. 2006;44(9):1140-1145.
